# Journey of young self-harm survivors from being vulnerable to resilient: Participants’ perspective on a culturally-adapted self-harm prevention intervention

**DOI:** 10.1192/j.eurpsy.2023.2367

**Published:** 2023-07-19

**Authors:** N. Chaudhry, S. Tofique, T. Kiran, I. B. Chaudhry, N. Husain, E. Colucci

**Affiliations:** 1 Research, Pakistan Institute of Living and Learning; 2 Psychiatry, Ziauddin Hospital, karachi, Pakistan; 3 Division of Psychology and Mental Health, University of Manchester, Manchester; 4 Department of Psychology, Middlesex University, London, United Kingdom

## Abstract

**Introduction:**

Repeated self-harm represents the single strongest risk factor for suicide. Worldwide, suicide is the second leading cause of death in young people aged 15-29 year, and the leading cause of death in many Asian countries.

**Objectives:**

This qualitative study was nested in a multi-center effectiveness trial of a **Y**outh **C**ulturally-adapted **M**anual **A**ssisted Problem-solving intervention (Y-CMAP) for prevention of self-harm in Pakistan and aimed to explore young people’s perspective on the intervention.

**Methods:**

One-to-one in-depth qualitative interviews were conducted with 20 participants from 5 cities across Pakistan, using a semi-structured topic guide to explore their views about self-harm, Y-CMAP intervention content, perceived effectiveness and challenges. Interviews were conducted in Urdu language, digitally recorded, transcribed verbatim and translated into English. Thematic analysis was conducted by the trained qualitative researchers.

**Results:**

Interpersonal conflicts including relationship difficulties, financial problems, and lack of social support were highlighted as precipitating factors of self-harm. Participants reported that Y-CMAP intervention is structured and easy to understand. They acknowledged the role of distraction techniques, cost-benefit analysis, discussion on thinking pattern, problem-solving and anger management in improving their mental health and wellbeing and reduce self-harm. Participants also shared their initial fears regarding the intervention, such as fear of disclosure of information to media. School and job timings were described as potential challenges for participation in the intervention.

**Conclusions:**

Exploring the perspectives of young people about culturally-appropriate intervention is imperative in their journey towards preventing suicide, which is a preventable cause of premature death. Findings are particularly relevant for Pakistan, one of the youngest nations in the world with limited resources for suicide prevention.

**Disclosure of Interest:**

None Declared

